# Toward a service-based workflow for automated information extraction from herbarium specimens

**DOI:** 10.1093/database/bay103

**Published:** 2018-10-08

**Authors:** Agnes Kirchhoff, Ulrich Bügel, Eduard Santamaria, Fabian Reimeier, Dominik Röpert, Alexander Tebbje, Anton Güntsch, Fernando Chaves, Karl-Heinz Steinke, Walter Berendsohn

**Affiliations:** 1 Botanic Garden and Botanical Museum Berlin, Freie Universität Berlin, Königin-Luise-Str. Berlin, Germany; 2 Fraunhofer-Institute of Optronics, System Technologies and Image Exploitation, Fraunhofer Str. Karlsruhe, Germany; 3 Faculty I—Electrical Engineering and Information Technology, Hannover University of Applied Sciences and Arts, Ricklinger Stadtweg, Hannover, Germany

## Abstract

Over the past years, herbarium collections worldwide have started to digitize millions of specimens on an industrial scale. Although the imaging costs are steadily falling, capturing the accompanying label information is still predominantly done manually and develops into the principal cost factor. In order to streamline the process of capturing herbarium specimen metadata, we specified a formal extensible workflow integrating a wide range of automated specimen image analysis services. We implemented the workflow on the basis of OpenRefine together with a plugin for handling service calls and responses. The evolving system presently covers the generation of optical character recognition (OCR) from specimen images, the identification of regions of interest in images and the extraction of meaningful information items from OCR. These implementations were developed as part of the Deutsche Forschungsgemeinschaft-funded a standardised and optimised process for data acquisition from digital images of herbarium specimens (StanDAP-Herb) Project.

## Introduction

### Herbarium collections

A herbarium is a collection of preserved specimens of plants, fungi and algae. Herbarium collections contain >350 million specimens worldwide (1). These specimens and their related data provide a huge amount of valuable and useful information: descriptions of taxa in floras and monographs are largely based on herbarium specimens. They document the distribution of taxa and often come with additional information, e.g. about the habitat or uses of the plant at the place of collection. Specimen vouchers make it possible to review identifications (2), thus allowing to falsify the assertion of their taxonomic classification as well as any conclusion based on that. They also serve as raw materials for several kinds of analyses, with molecular techniques like generating deoxyribonucleic acid (DNA) sequences becoming increasingly important. They serve as reference material for a number of fields of biological research ranging from systematics to ecology and biodiversity. In particular, through their long history, herbarium collections document changes of plant biodiversity over time and in space. For example, with the help of specimen information, it became possible to provide substantial proof for the assumptions that and how temperature changes cause shifts in geographic distributions and flowering times (3). Herbarium specimens were also used to show how a change in air quality affects the plants and to trace movements of invasive species (3).

**Figure 1:**
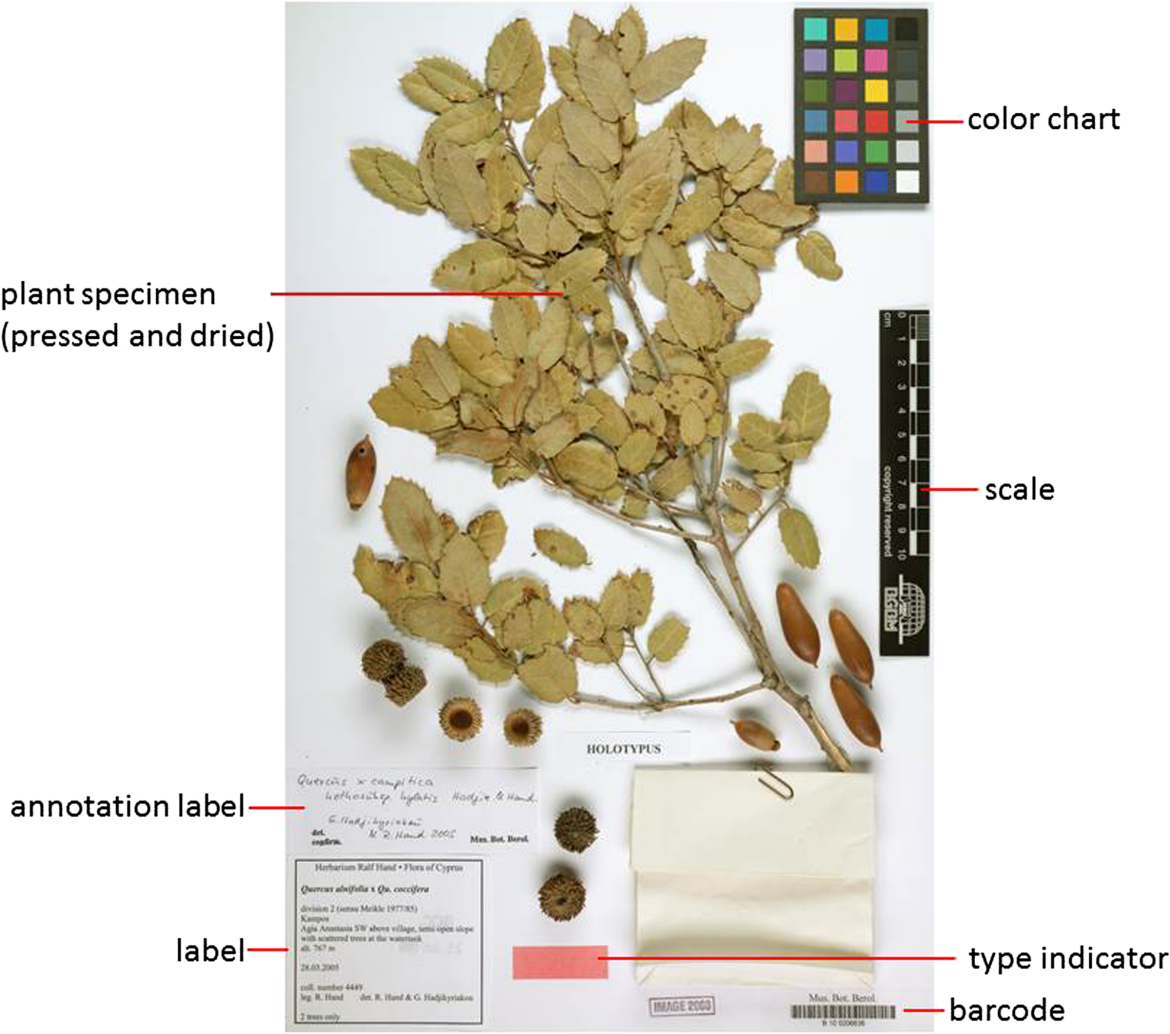
Herbarium sheet.

### Information on plant specimens

**Figure 2:**
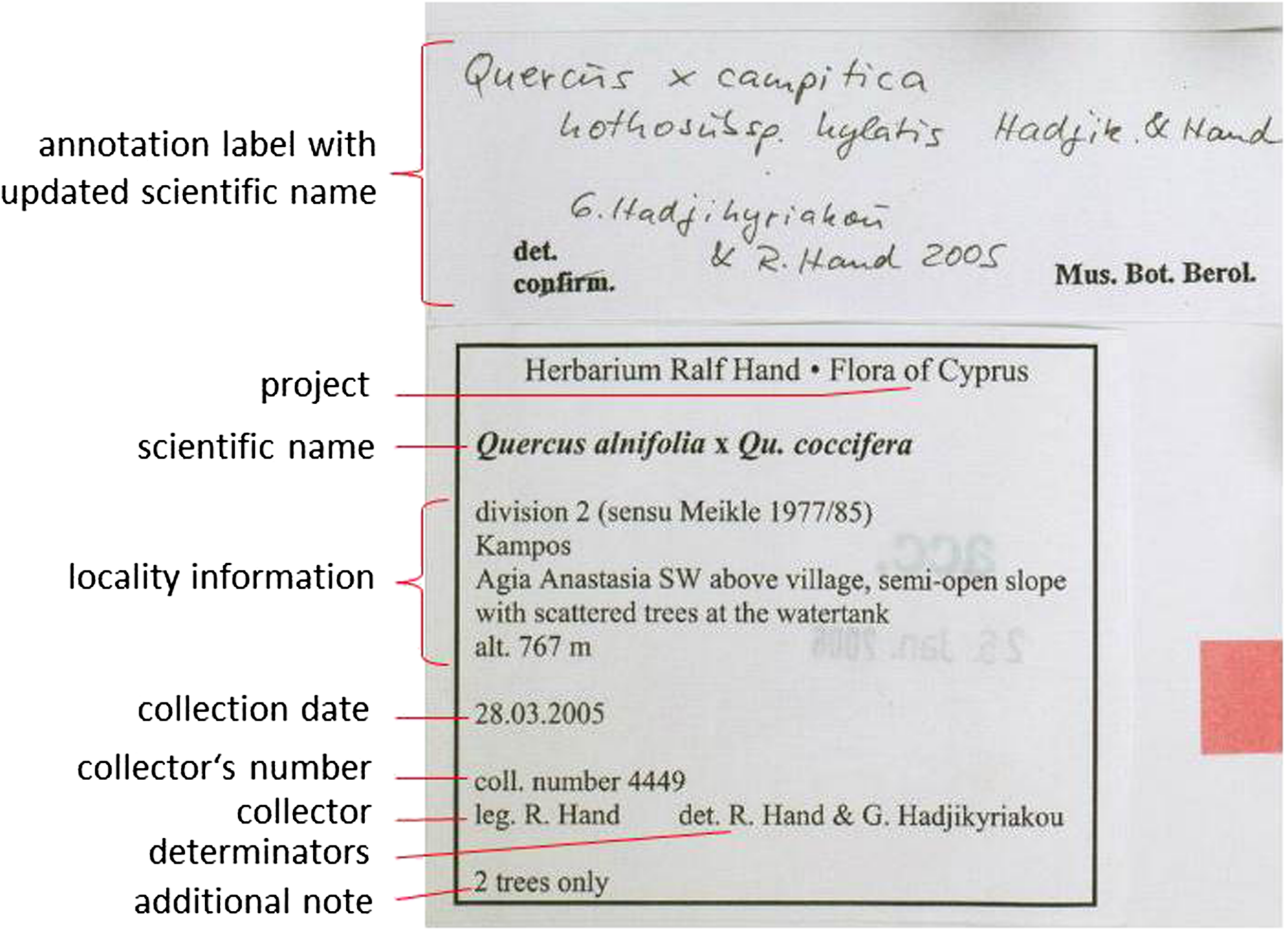
Information on specimen labels.

The method of preserving vascular plants has hardly changed since its introduction 500 years ago (1): collected plants are pressed and dried and then fixed on paper sheets. The specimen is labeled with information like the scientific name of the plant, place and date of collection and the name of the collector and/or the name of the project or collecting event, a collection number, name of the determiner and notes on the habitat and habit of the plant. In some cases, the label is supplemented by further labels, for example correcting or amending the original scientific name (annotations) (Figures 1 and 2).

The scientific name of a species or infraspecific taxon refers to a nomenclatural type, i.e. normally a single specimen designated when the taxon was first described and named. Type specimens are often marked by a red rectangle or the word `type’ stamped on the sheet.

In preparation of the photographic recording of a specimen, a scale, and often a color chart, is mounted on the paper sheet in order to put the size and color spectrum of the specimen image in relation to the original specimen (4) (Figure 1).

**Figure 3:**
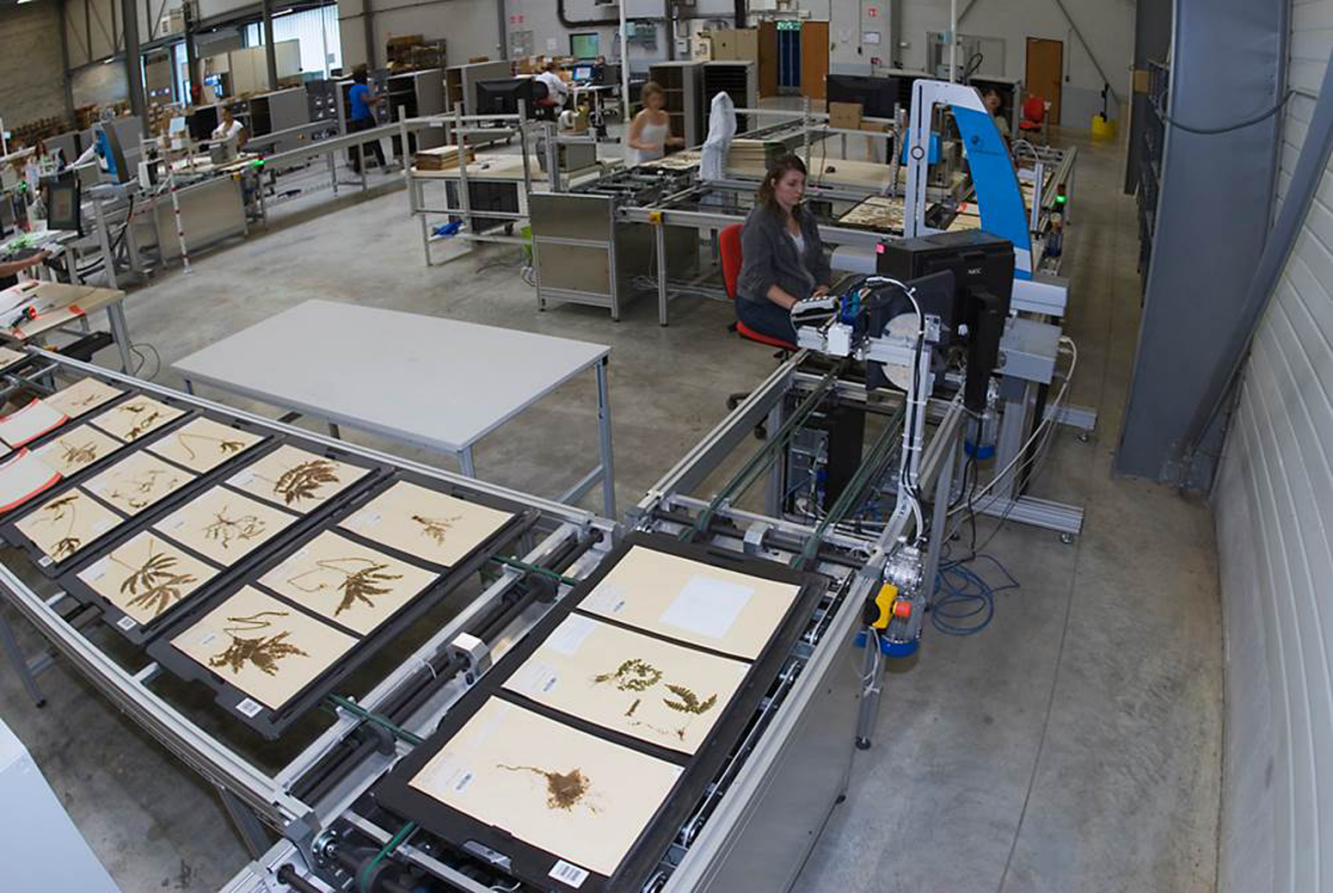
Digistreets for the MNHN in Paris (photo: P. Lafaite, MNHN).

### Herbarium data for research

In the following, the information located on the herbarium vouchers, in particular the information on the labels, is referred to as `data’ (5, 6). The term metadata is used in the sense of Meon *et al.* (7) to designate transformed, enhanced and structured data. Databased specimen metadata allow wide-ranging searches and analyses of biodiversity information based on herbarium sheets. Search results serve for instance as a basis for the generation of time series and species distribution maps showing changes of species composition over time [e.g. (8, 9)].

Especially for cross-disciplinary research questions such as those related to climate change, all available metadata should be utilized. International information networks like the Global Biodiversity Information Facility (http://www.gbif.org) and the Biological Collection Information Service (10) (http://www.biocase.org) publish freely available biodiversity data according to common standards such as Access to Biological Collection Data (ABCD) (11) and Darwin Core (DwC) (12), providing joint access to collection databases worldwide.

### Mass digitization and data capture

Over the past years, several natural history institutions started industrial scale digitization processes for their specimen holdings.

Between 2009 and 2012, 8 million herbarium specimens from the Muséum National d’Histoire Naturelle (MNHN) in Paris were digitized for the first time using a conveyor belt approach (Figure 3). The `Digitarium’ in Joensu, Finland, set up a similar procedure (13) with ∼7 million objects (also from zoology) being digitized. In 2010, the Natural History Museum `Naturalis’ in the Netherlands started a program for mass digitization (14) and digitized 4 million herbarium specimens in 1.5 years.

As a result of this production-line digitization, a large throughput is achieved in the photographic recording of specimens. However, the manual capture of the data remains a bottleneck. At the same time, data capture a prerequisite for accessing and cross-linking the collections with their related information.

**Figure 4:**
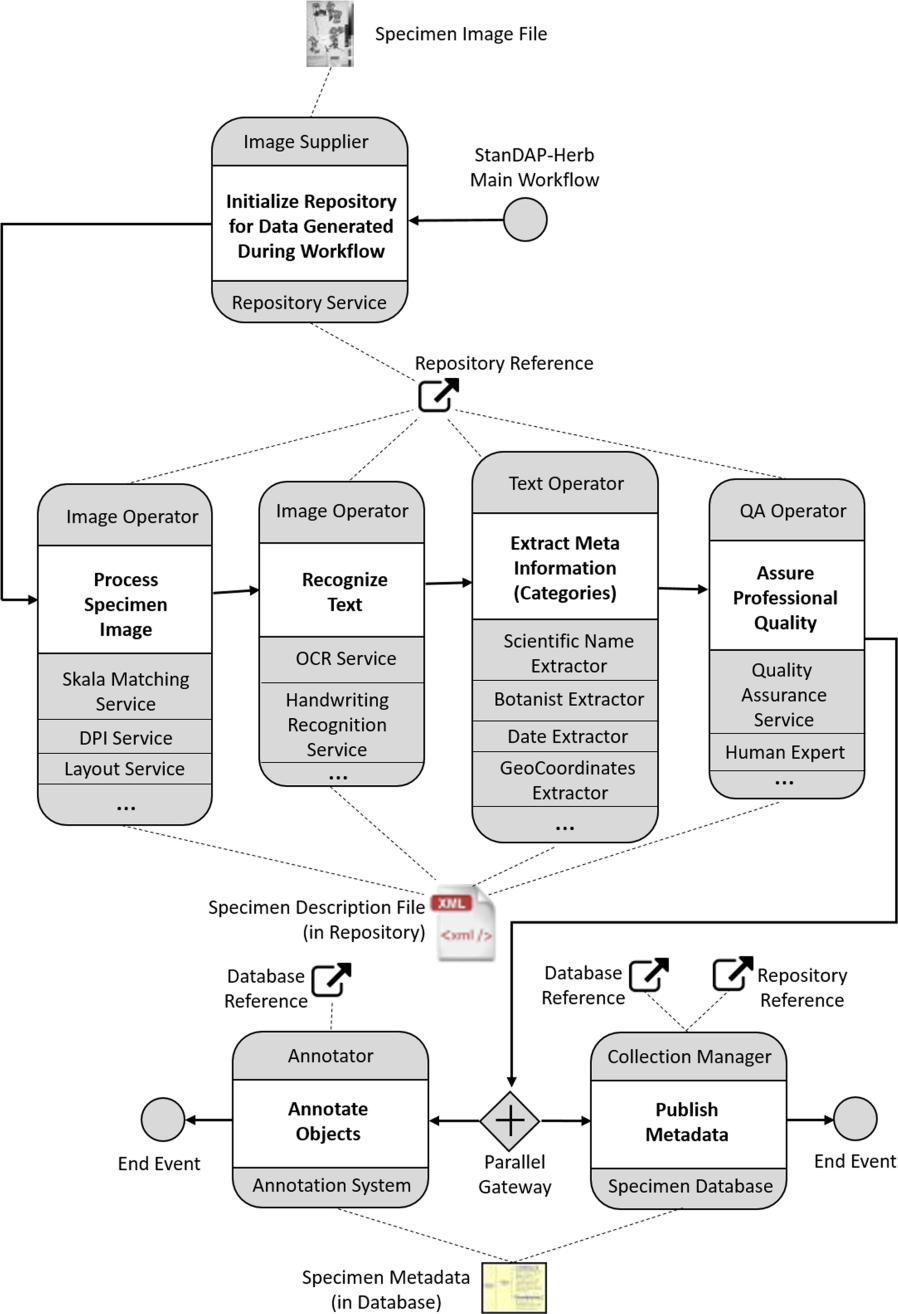
The StanDAP-Herb main choreography.

### Automatic data capture

There are already a number of individual tools and services for automated data extraction from images available, but the functionality covered is incomplete and/or cannot be integrated via standard interfaces. We investigated such systems, but they did not fit in with our basic idea of an extensible open-source web service-based workflow platform. Symbiota (15) is a complete Herbarium image management software with additional functions to automatically extract data, rather than an extensible open source Application Programming Interface (API) for automatic data extraction. The Salix parser (16) is a stand-alone tool used to extract and classify information from text sequences and could be added as a service to our workflow system but does not cover other functionalities needed for a complete workflow. The apiary project which was focused on parsing specimen label data is not available any more. Up to now, there is no integrated and extensible workflow platform available which combines services and tools provided by the biodiversity informatics community (see Workflows and software architecture section below). With the aim of speeding up the data capture process, the StanDAP-Herb project developed a standardized workflow comprising web service modules, which carry out a stepwise (partly) automated capture of data contained in herbarium images, as described in the following.

## Workflow

In this section, the concepts of services and workflows are outlined on which the specifications and development work documented in Workflows and software architecture and Outlook sections are based.

The StanDAP-Herb architecture is based on standardized interfaces and established IT standards as well as on a platform-independent technical specification, which can easily be adapted to specific platforms. This is to facilitate a long software life cycle as well as an easy integration of and into systems operated by third parties and to allow globally interacting services in workflows defined across organizational boundaries.

Figure 4 outlines the StanDAP-Herb main workflow choreography, which is described as a set of interacting choreographies that constitutes the ‘mainstream' processing of a digitized specimen. We choose a choreography diagram (17) because it is the adequate means for describing the global communication across enterprise boundaries. It describes the agreements between the main interacting participants with respect to documents, message types and sequences of message flows. The internal view of the processes is then further outlined by means of Business Process Model and Notation (BPMN) diagrams (17), which have been developed in the project and are partially outlined in Workflows and software architecture section.

A choreography task represents the interaction between two participants. The participant initiating the communication (e.g. `Image Supplier’ as shown in Figure 4 the uppermost choreography task) is placed above the entire choreography task (here: `Initialize Repository’). The receiving participant (here: the `Repository Service’) is shown below the choreography task. The Image Supplier is assumed to be the owner of a graphics file representing a digitized herbarium specimen (BPMN artifact `Specimen Image File’). The Repository Service is an instance that creates a global reference by means of which the file can be retrieved for further processing. The range of mechanisms for generating this reference may vary concerning the organization providing this service; for instance, the repository reference could lead to a cloud service where the specimen description files (SDFs) are stored.

By means of the repository reference, a participant named `Image Operator’ can retrieve the Specimen Image File and conduct some analytic work on the image. This work is executed by services that directly work on the image, e.g. extraction of the scale, identification of dots per inch (DPI) or location of text areas (cf. the image-based services used in the `Pre-OCR Workflow’ defined in Workflows and software architecture section). The Image Operator adds the results computed by these services to the SDF, which is augmented during the process by gaining the results of each stage. In a further step, the Image Operator starts the processes for character recognition; in addition, to the optical character recognition (OCR) services working on machine printed text, services for handwriting recognition would be needed here.

In the next choreography task, the focus is on further elaboration of the texts which have been found on the herbarium specimen by the text recognition services. Besides information extraction in order to find categories such as the taxon, the collector or the owner of a herbarium sheet, a further task is to complete incomplete texts by means of tools for auto-completion or manual completion. The `Text Operator’, who conducts and coordinates all text processing activities, should thus be assisted by means of a proper user interface.

Data quality assurance is the topic of the next task (`Assure Professional Quality’); two basic mechanisms are distinguished:

(i) Machine-based approaches: platforms such as `Open Refine’ (18) can be used to harmonize many faceted information collected in the SDFs.
(ii) Human-based approaches: in certain cases, expert knowledge may be needed in order to resolve conflicting statements or missing information; experts such as taxonomists are to be contacted in order to review and pass an expert opinion back to the operator. The Quality Assurance (QA) operator will then incorporate corrections and comments given by the specialists into the SDF.

Of course, both approaches may be combined interactively and iteratively. An example for the application in a workflow environment was set by the European Union (EU)-funded Biodiversity Virtual e-laboratory (BioVeL) project (19).

When the object and category files are complete and quality assured, they will be handed over to the `Collection Manager’, for whom the data have been investigated. The Collection Manager (or the `Curator’, respectively) can then store the metadata in a specimen database for publication. For this purpose, the metadata is to be converted into a standard format for biological collection data (ABCD or DwC). Before incorporating and releasing the metadata record, it is matched against existing data (in the local specimen database or in the network) and in case duplicates are found, the new metadata and the found duplicate have to be merged. For example, if a duplicate in the network contains additional information, this can be added to the metadata before generating the new database entry.

**Table 1:** Web services to enable the implementation of workflows for processing digital herbarium specimens (23, 24)

**Category**	**Subcategory**	**Name**	**Input**	**Output**	**Description**
Image-based	Object recognition	Scale Matching Service	GUID, SRI	Scale region coordinates	Template matching algorithm (uses an example image of the searched for scale to detect it in other images) (23)
Image-based	DPI recognition	DPI Service	SRI resolution, SRI size, scale region coordinates	DPI	Computation of DPI using the physical size of the actual scale and the size of its digital counterpart on the herbarium sheet
Image-based	Object recognition	Text Region Service	GUID, DPI	Text region coordinates	Line contrast approach (taking advantage of the fact that the horizontal contrast of text lines is very high and dark and bright areas are alternating quickly)/machine learning approach to detect text-like structures
Image-based	OCR	Tesseract/OmniPage Service	GUID, text region coordinates	Text	Tesseract/OmniPage OCR algorithm
Text-based	Dictionary-based	Scientific Name Extractor	Text	Scientific names	Parsing with dictionary based on Global Names (http://gnrd.globalnames.org/) and WikiData (24)
Text-based	Dictionary-based	Botanist Name Extractor	Text	Botanist names	Parsing with dictionary based on botanists’ database of the Harvard University Herbaria & Libraries (http://kiki.huh.harvard.edu/databases)
Text-based	Regular expression	Date Extractor	Text	Dates	Matching using regular expressions for dates related to collection, accession and determination
Text-based	Regular expression	Geographical Coordinates (GeoCoord) Extractor	Text	Coordinates	Matching using regular expressions for geographical coordinates
Text-based	Dictionary-based	Location Extractor	Text	Locations	Parsing based on Cartographic Location and Vicinity Indexer (CLAVIN) library (http://clavin.bericotechnologies.com)

GUID indicates Globally Unique Identifier; SRI, Scale Reference Image.

As soon as the metadata set is stored in the specimen database, it can be annotated, i.e. further data or changes to the metadata may be made. This can be done by the Collection Manager or Curator but also by other actors using the public data access. For the latter, an external `Annotation System’ has to be used [e.g. AnnoSys, (20, 21)], which stores annotations made from all online portals where a metadata record set from a specimen database has been published. In our workflow, we are also assuming that Collection Managers can be informed as soon as annotations have been made in an Annotation System and that they verify these annotations and eventually incorporate them into their specimen database.

Since data curation of imaged collections poses a challenge to personnel resources of herbaria, an alternative procedure (in agreement with the Image Supplier) could be to store the gained metadata directly in an annotation repository like AnnoSys, i.e. to treat them as annotations to the original metadata. This permits an asynchronous processing of the specimen data, i.e. they may become accessible immediately. However, full use of temporarily or permanently stored extraction results—as annotations in data-driven research workflows—requires the integration of data access services for these annotations into the infrastructures of the corresponding data portals.

## Services

We developed several web services to enable the implementation of workflows for processing digital herbarium specimens. Some services were developed from scratch; others are using third-party libraries and wrap already existing functionalities to make them available for our workflows. In Table 1, all available services are listed.

Image-based services are working on specimen image data. They have two goals. The first one is to optimize the results of data extraction by locating objects on herbarium images which provide information about the specimens. The second one is to pre-sort herbarium images by criteria like `possibility of automatic processing’ or `handwritten label’.

Text-based services are working on text parts extracted by image-based services. They can recognize a set of entities like plant name, collector name, geographical coordinates and/or mentioned locations. These services use either dictionaries or regular expressions (22) for entity recognition.

**Figure 5:**
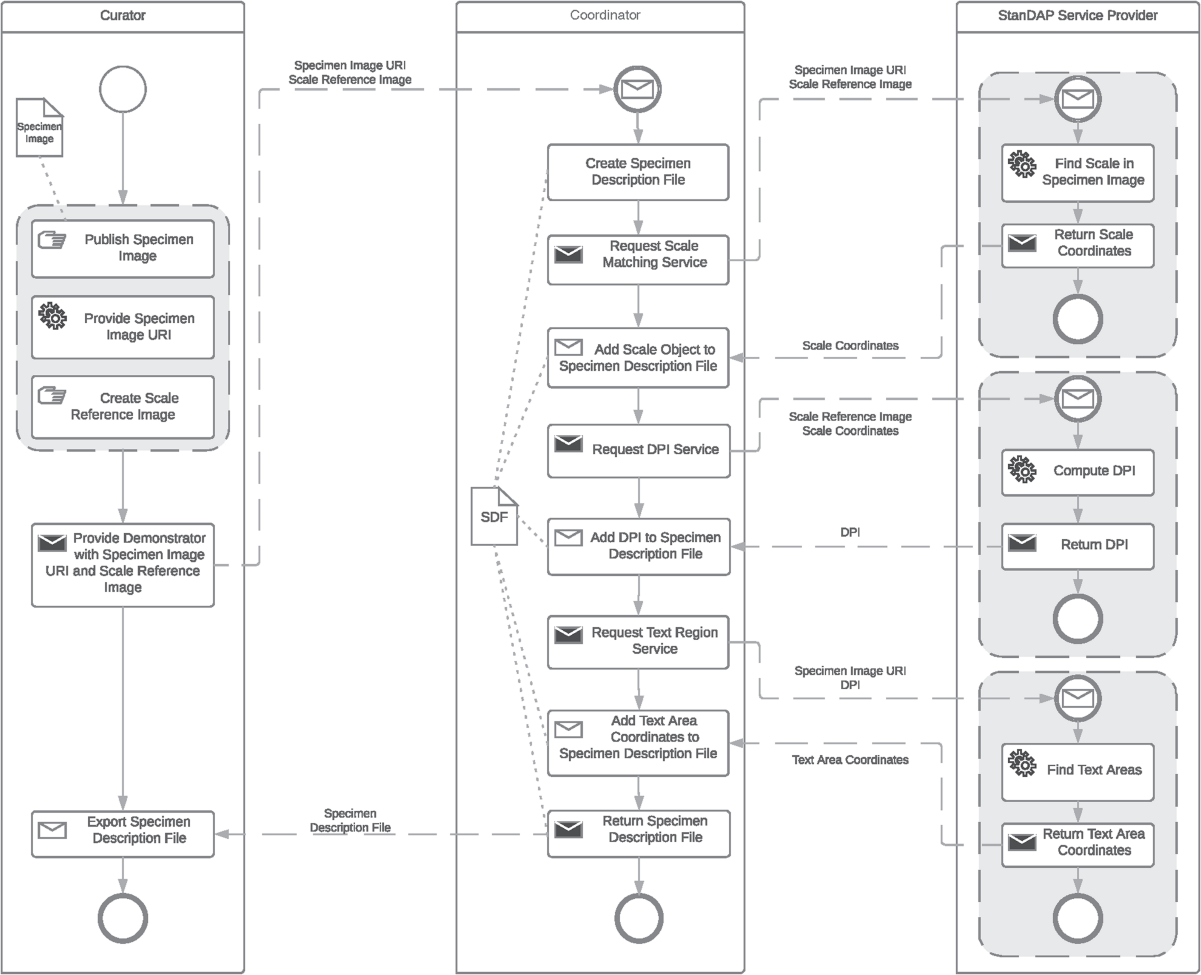
Pre-OCR workflow.

**Figure 6:**
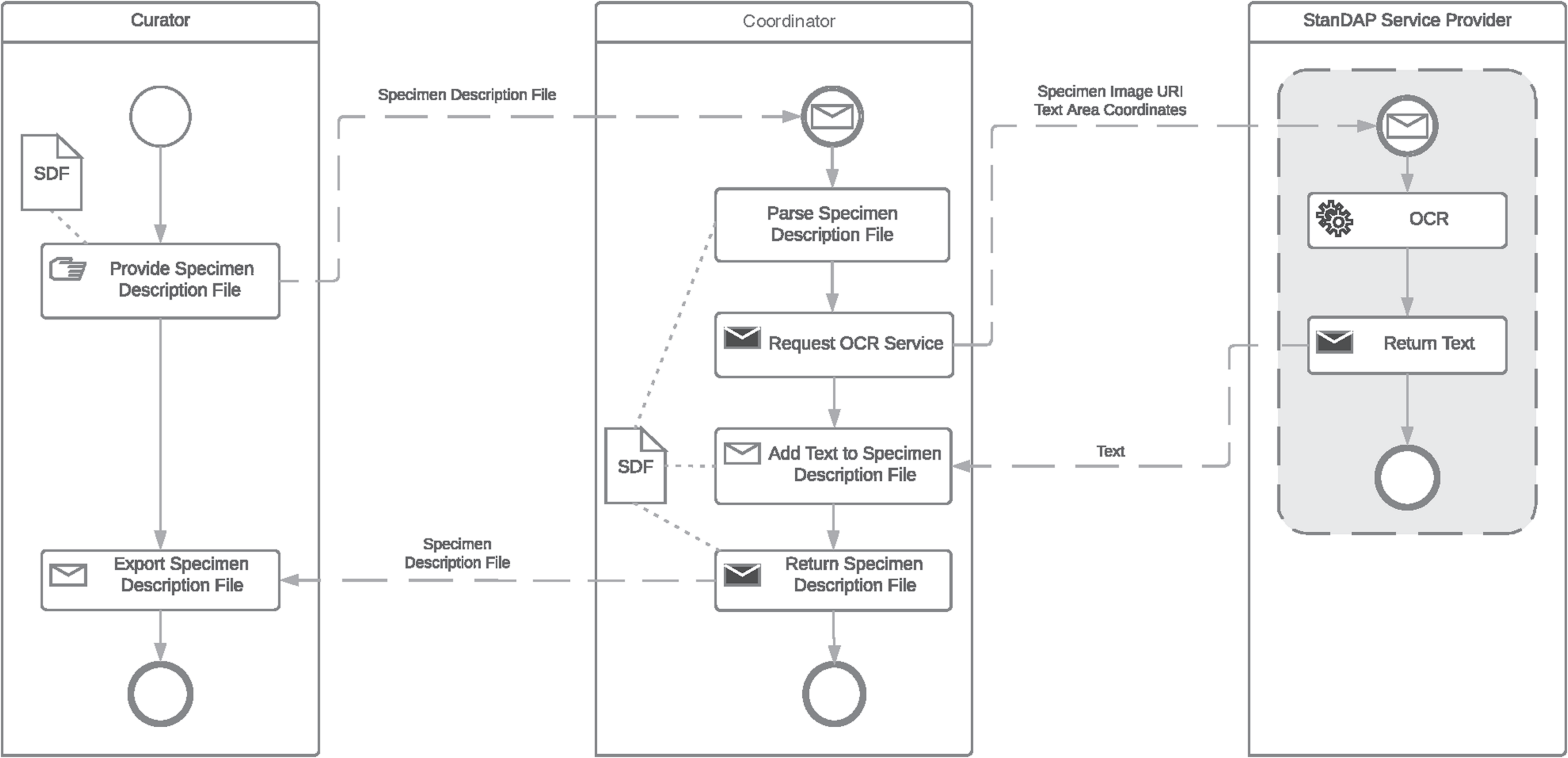
OCR workflow.

To build a workflow, it is essential to know what a service requires as input and what can be expected as output. As long as required input and given output type of a service stay the same, the exact procedure to get from the input to the output can be changed without influencing other parts of already configured workflows. Table 1 shows that the output of some services can be used as input for others and that it is therefore possible to build chains of services to process.

The documentation of the developed services is available using a Swagger framework (https://swagger.io/) instance under http://api.bgbm.org/standap/v1/doc.

## Workflows and software architecture

### Example workflows

To show the potential of the developed web services, three example workflows were developed. In the following, these will be briefly described. All of them are based on three actors: the image supplier, the coordinator and the service provider. An example for an image supplier would be an institute that wants to analyze one of its digital herbarium sheets. The image supplier interacts with the coordinator whose function is executing an analysis pipeline by requesting different services provided by the service provider, aggregating the results and returning them to the image supplier. An extension for the open refine platform, supporting the execution of the pipelines here described and fulfilling the role of the coordinator was developed and can be downloaded under http://api.bgbm.org/standap/download/openrefine-extension.

### Example 1: Pre-OCR Workflow

The OCR is one of the most important steps to extract data from digital specimen images. It is therefore crucial to optimize it to get the best possible results. The aim of the Pre-OCR Workflow is to compute the DPI of a given digital specimen image and find text areas within that image. These data can then be used to improve OCR results by letting the OCR software just process the identified text regions and therefore reduce noise, such as parts of the plant specimens that are interpreted as letters. DPI recognition is important because most OCR engines are specialized on processing images of a specific DPI. The restriction of the OCR process to areas of actual text also reduces the execution time.

Three services are used to create the Pre-OCR Workflow:

***Scale Matching Service.*** It uses template matching to compute the coordinates of the scale (see Figure 1) of a specimen image.

***DPI Service.*** It is able to compute the DPI of a specimen image out of the scale coordinates computed by the Scale Matching Service and the resolution and size of the SRI used in the Scale Matching Services template-matching process.

***Text Region Service.*** It can find the coordinates of text areas in specimen images.

In the following, it will be assumed that the workflow is started by an Image Supplier as the user who publishes the specimen images to get processed by the Pre-OCR Workflow and provides an SRI to the coordinator. An SRI is an image of the scale mounted on the analyzed specimen. For unique specimen image Uniform Resource Identifiers (URIs), we make use of a consistent system of Globally Unique Identifiers (GUIDs) agreed by the Consortium of European Taxonomic Facilities (25, 26). The user provides the GUID(s) to the coordinator, which then first creates an SDF for each GUID, in which all information gained throughout the execution of the workflow will be saved. Next the Scale Matching Service is requested with each of the given GUIDs and the configured SRI as parameters. The resulting coordinates in combination with the known resolution of the SRI and the physical size of the scale are then used to request the DPI Service. With the returned DPI numbers, it is possible to invoke the Text Region Service, which responds with the coordinates of text areas found in the specimen images referenced by the given GUIDs. All results of the services are available in the SDFs, which are provided to the user by the coordinator at the end of execution. In the following, these files can be exported and saved for later usage, e.g. to start the OCR Workflow described in the next section. The BPMN of the Pre-OCR Workflow is shown in Figure 5.

**Figure 7:**
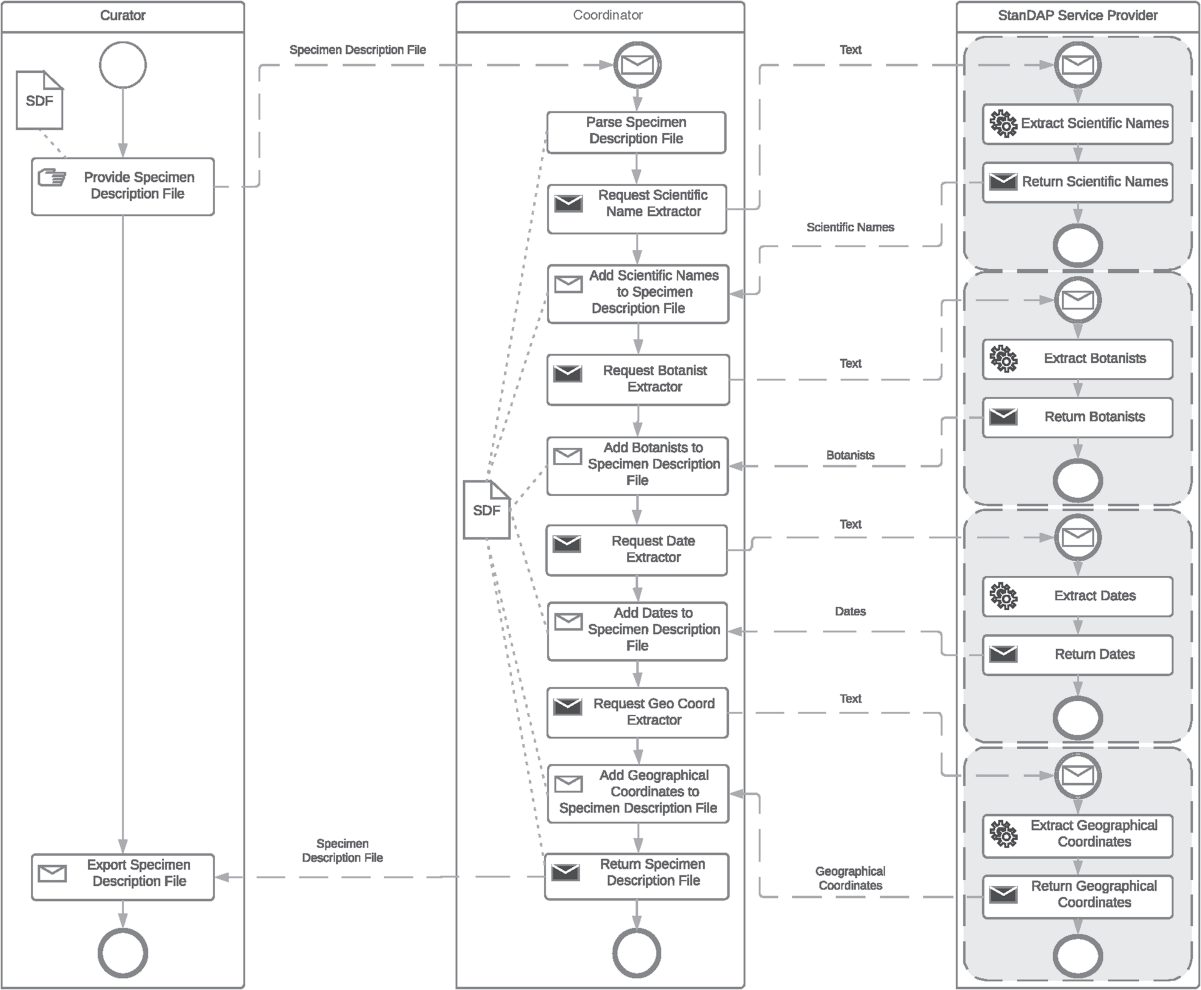
Extractor workflow.

### Example 2: OCR workflow

The OCR workflow is used to find and read text located on a digital specimen image, which will then be analyzed to extract metadata. It is started by the user who generated the Specimen Description File (SDF) using the pre-OCR workflow as detailed above. The user can feed in several SDFs at once. The coordinator parses the description file and requests the OCR service with the GUID and the coordinates of text areas previously found by the Text Region Service. The returned text parts are added to the SDF which is then returned to the user. The BPMN of the OCR Workflow is shown in Figure 6. For this approach, it is necessary that the requested OCR service supports the processing of image parts by coordinates.

### Example 3: Extractor workflow.

The extractor workflow combines all text-based extractor services and thus enables the extraction of a maximum amount of metadata from a given text. The following services are available for the text provided:

***Scientific Name Extractor.*** It finds and parses scientific names.

***Botanist Name Extractor.*** It finds and parses botanist names.

***Date Extractor.*** It finds and parses collection, accession and determination dates.

***GeoCoord Extractor.*** It finds and parses latitude and longitude pairs.

***Location Extractor***. It finds and parses location information and assigns a country.

The workflow is once more started by the user providing SDF(s). All Extractor Services are then requested with the text parts parsed from the SDF. The order of requests sent by the coordinator is not important. Figure 7 gives an example workflow.

## Outlook

A key aim of the StanDAP-Herb project was the investigation of possibilities for combining a service-oriented architecture (SOA) with the advantages of a flexible workflow management system. As outlined in Outlook section, this flexibility is needed for handling changing requirements such as

(i) managing entry points at which external organizations can use (parts of) the StanDAP-Herb workflow,
(ii) enabling external organizations to integrate their services into the StanDAP-Herb workflow chain and
(iii) providing a high degree of automation whilst easily permitting manual interaction at specific stages.

Many of the workflow systems that have been investigated like Taverna (27), Kepler (28) and Argo (29) do not fulfill the StanDAP-Herb requirements in one or more respects. Some lack the possibility to describe complex workflows in a standardized notation such as BPMN; others do not offer a comprehensive run-time support for integration of services in the context of an SOA.

As a result, we identified the Activiti workbench (http://www.activiti.org/) as the most promising system. Activiti can be used as a library or a service; it provides a number of tools for design and deployment of complex workflows, e.g. graphical representations of workflows, support of BPMN 2.0 constructs, integration of user management and specification of forms in the workflow definition. The Activiti service interface can be used to deploy a process.

However, as with other tools, it is necessary to write Java classes for that purpose. At present, a solution that can provide full integration of SOA by configuration is to our knowledge not available. Consequently, one of the main StanDAP-Herb ideas can only be realized by development of more sophisticated background technology. Future research and development should focus on this item.

To make the created web services available for a larger user base and for testing purposes, the OpenRefine extension mentioned above was developed, offering easy access to the basic usage of the described web services as well as access to Herbadrop (30) OCR results. Further development of the extension is necessary to increase the usability of the described work.

The requirement of easy integration of third party services into the StanDAP-Herb installation mandates direct usage of available registries for biodiversity services such as the `Biodiversity Catalogue’ (http://www.biodiversitycatalogue.org/). Principally, such web-based services offer Representational State Transfer (REST) or originally Simple Object Access Protocol (SOAP) interfaces (the trend is going to the REST interface that is easier to use). For future applications, we recommend usage of message-oriented middleware (MOM) technology (31) that can provide `publish/subscribe’ schemata. Moreover, a MOM broker such as RabbitMQ (https://www.abbitmq.com/) or QPID (https://qpid.apache.org/) can provide queue mechanisms for delivery and processing of messages and thus directly support connection of external organizations to a StanDAP-Herb workflow installation.
